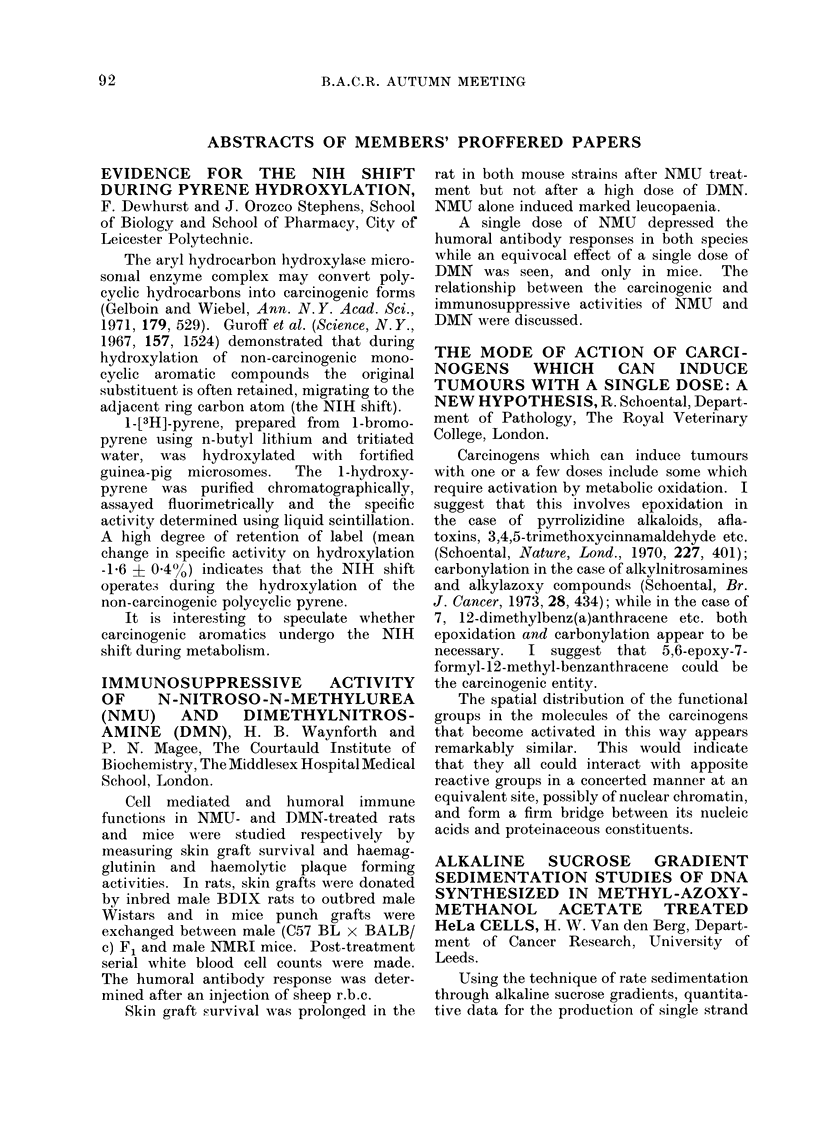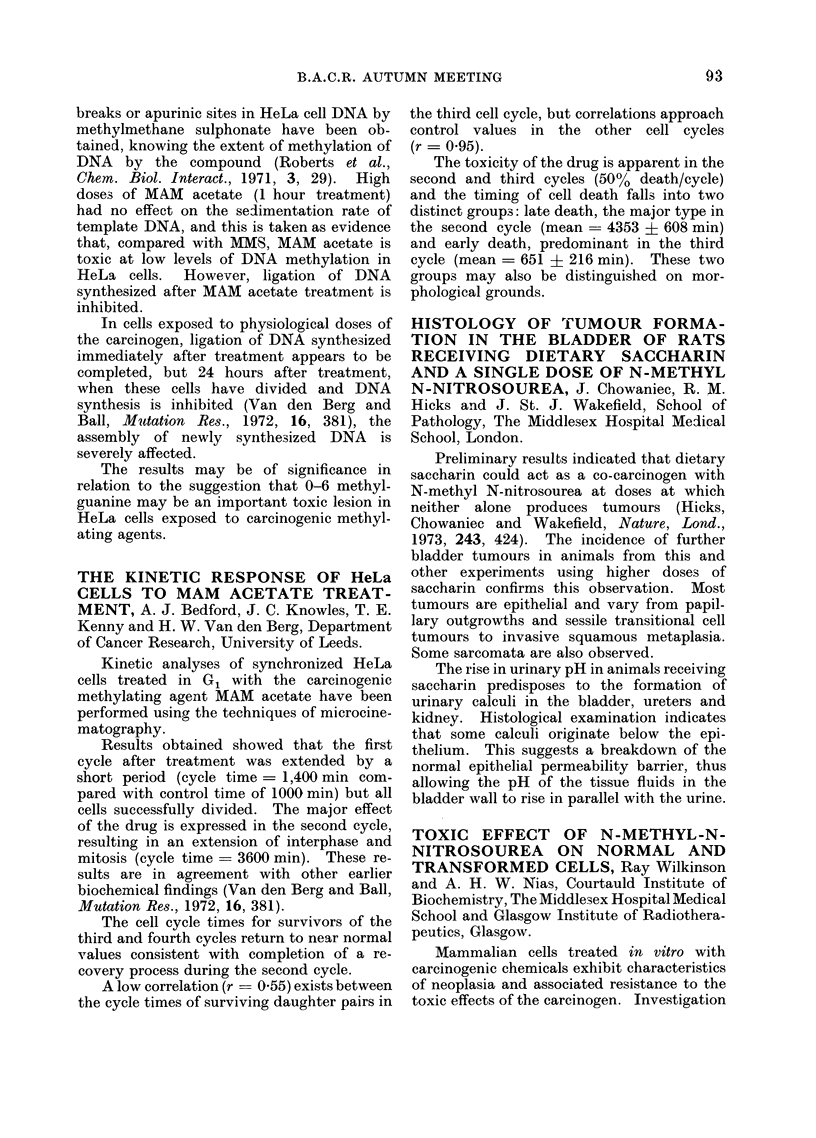# Proceedings: Alkaline sucrose gradient sedimentation studies of DNA synthesized in methyl-azoxymethanol acetate treated HeLa cells.

**DOI:** 10.1038/bjc.1974.16

**Published:** 1974-01

**Authors:** H. W. Van den Berg


**ALKALINE SUCROSE GRADIENT SEDIMENTATION STUDIES OF DNA SYNTHESIZED IN METHYL-AZOXYMETHANOL ACETATE TREATED HeLa CELLS,** H. W. Van den Berg, Department of Cancer Research, University of Leeds.

Using the technique of rate sedimentation through alkaline sucrose gradients, quantitative data for the production of single strand breaks or apurinic sites in HeLa cell DNA by methylmethane sulphonate have been obtained, knowing the extent of methylation of DNA by the compound (Roberts *et al.*, *Chem. Biol. Interact.*, 1971, **3**, 29). High doses of MAM acetate (1 hour treatment) had no effect on the sedimentation rate of template DNA, and this is taken as evidence that, compared with MMS, MAM acetate is toxic at low levels of DNA methylation in HeLa cells. However, ligation of DNA synthesized after MAM acetate treatment is inhibited.

In cells exposed to physiological doses of the carcinogen, ligation of DNA synthesized immediately after treatment appears to be completed, but 24 hours after treatment, when these cells have divided and DNA synthesis is inhibited (Van den Berg and Ball, *Mutation Res.*, 1972, **16**, 381), the assembly of newly synthesized DNA is severely affected.

The results may be of significance in relation to the suggestion that 0–6 methylguanine may be an important toxic lesion in HeLa cells exposed to carcinogenic methylating agents.